# Salt-sensitive hypertension: role of endothelial and vascular dysfunction and sex

**DOI:** 10.3389/fphar.2025.1565962

**Published:** 2025-03-12

**Authors:** Helen M. Butler, Marice K. McCrorey, Lada Palygina, Ryan Lacey, Justin P. Van Beusecum

**Affiliations:** ^1^ Division of Nephrology, Department of Medicine, Medical University of South Carolina, Charleston, SC, United States; ^2^ College of Graduate Studies, Medical University of South Carolina, Charleston, SC, United States; ^3^ Ralph H. Johnson VA Healthcare System, Charleston, SC, United States

**Keywords:** sex differences, salt-sensitive blood pressure, endothelial dysfunction, vascular, endothelial cell (EC)

## Abstract

For the last 120 years, the contribution of salt has been identified in the pathophysiological elevation of blood pressure. Since then, both human and experimental murine studies have begun to elucidate the key mechanisms contributing to the development of salt-sensitive hypertension. Numerous mechanisms, including increased plasma volume, sodium retention, impaired autoregulatory capability, inflammation, and endothelial and vascular dysfunction, contribute to deleterious elevations in blood pressure during salt sensitivity. The endothelium plays a critical role in blood flow regulation, renal blood flow, and blood pressure elevations and in migrating immune cells to end-organs, contributing to end-organ damage and fibrosis. In this review, we will consider the clinical studies setting the foundation for the definition of salt-sensitive hypertension, murine models to study endothelial and vascular contributions, and endothelial cell cultures that have shed light on signaling mechanisms. Lastly, we will discuss the sex-dependent physiology and mechanisms contributing to salt-sensitive hypertension development and their clinical implications.

## Introduction

Hypertension, classified by blood pressure above 120 mmHg systolic or 80 mmHg diastolic, is the leading risk factor for cardiovascular morbidity and mortality, is present in more than 30% of adults globally, and has associations with age and sex (Mills et al., 2020; NCD Risk Factor Collaboration NCD-RisC, 2021). Multiple contributing factors, such as lifestyle, genetics, and pharmacological intervention play a role in regulating blood pressure status. However, perhaps the most influential and modifiable variable is salt intake (Vogt et al., 2023; WHO, 2012). Salt-sensitive hypertension (SSHTN) is characterized by elevated blood pressure in response to dietary increases in salt. Around 30% of the population is considered salt-sensitive (Bailey and Dhaun, 2024; Chen, 2010), which poses a significant health challenge due to its contribution to cardiovascular disease (Mattson et al., 2021). Importantly, SSHTN disproportionately affects specific demographic groups, highlighting the interplay of environmental, socioeconomic, and genetic factors in pathophysiology. Among the most affected are African Americans, who have a higher prevalence of salt sensitivity (Morris et al., 1999), chronic kidney disease (CKD) (Hsu et al., 2003), and other related cardiovascular events (Arun et al., 2024). These demographic disparities drive the need for strategies to mitigate SSHTN and its complications.

Mechanistically, the vasculature has an apparent role in driving the pathogenesis of SSHTN, including reduced compliance, endothelial dysfunction, fibrosis, and oxidative stress (Wu et al., 2014; Loperena et al., 2018; Ferreira et al., 2021; Gao et al., 2023; Miyoshi et al., 1997). Further, SSHTN is largely associated with an immunological response, including increased immune cell propagation and inflammation (Li et al., 2014; Kirabo et al., 2014; Wenzel et al., 2021; Navaneethabalakrishnan et al., 2024). Crucially, immune cell infiltration in SSHTN can contribute to the pathophysiology of stroke, heart failure, and chronic kidney disease. While high dietary sodium chloride (NaCl) intake and salt sensitivity levels promote vascular and immunoinflammatory responses and end-organ damage, the direct cellular network(s) and mechanism(s) remain to be elucidated.

While numerous mechanisms contribute to the development of SSHTN, this review will focus on the vaso-dysfunction theory of SSHTN that has developed from human studies and the intricate mechanisms underlying salt-sensitive vascular dysfunction, including endothelial dysfunction, activation, and inflammation. We also discuss the apparent sex differences in salt sensitivity for the management of SSHTN.

## Clinical studies of salt-sensitive hypertension and vascular dysfunction

Large-scale associations between salt intake and blood pressure were first published by Dahl and Love, wherein an increase in hypertension prevalence was documented in individuals who self-reported high salt intake relative to those who did not add salt to their diets (Dahl and Love, 1954). Since then, patient studies of SSHTN have demonstrated that the phenotype of salt sensitivity in humans is a continuous response. For example, the heterogeneity in blood pressure responses to altered intake of dietary NaCl was reported in 1978 when hypertensive patients were given progressively increased NaCl levels over a week (Kawasaki et al., 1978). While salt sensitivity was delineated as a ≥10% increase in blood pressure, patients displayed a spectrum of responses to salt intake, suggesting that thresholds might misalign patient pathophysiology between salt sensitivity and resistance. However, sodium retention and weight gain further supported the blood pressure thresholds defining salt-sensitive patients from salt-resistant individuals.

Since then, methodologies ushered into the field by Weinberger and colleagues involved measurements of blood pressure after salt loading and depletion in human patients, which have lent mechanistic insights (Miyoshi et al., 1997). While various techniques have since been used to assess blood pressure in response to changes in dietary salt consumption, they have all produced reproducible and conforming results (Weinberger, 1996; Weinberger and Fineberg, 1991; Weinberger et al., 1993).

Those early studies formed the basis for the traditional, nephrogenic model of SSHTN, which posits that increased dietary NaCl in salt-sensitive individuals leads to an abnormally large renal retention of sodium, plasma volume expansion, a temporary increase in cardiac output, and a prolonged increase in systemic vascular resistance. However, later patient studies have found that vascular dysfunction could act as a causative pressure effect in developing SSHTN (Sullivan et al., 1987; Draaijer et al., 1993; Schmidlin et al., 2007; Schmidlin et al., 2011; Laffer et al., 2016; Mark et al., 1975). Thus, the concept of vascular dysfunction in SSHTN hypothesizes that total vascular resistance, rather than cardiac output, determines an individual’s blood pressure in response to dietary salt load. Vasodilation and a resulting decrease in peripheral resistance would form the normal vascular response to increased dietary salt, allowing salt excretion without an excessive rise in blood pressure. In the case of salt-sensitive patients, this vasodilation is blunted, and total peripheral resistance does not fall, thus resulting in a rise in blood pressure.

A hemodynamic study that assessed over a hundred normal and borderline hypertensive patients during periods of salt depletion and repletion discovered that the subset of patients determined to be salt sensitive due to a rise in blood pressure during NaCl repletion had significant elevations in forearm vascular resistance (Sullivan et al., 1987). These results mirrored findings of more than a decade prior, wherein normotensive salt-resistant individuals had the appropriate lowering of forearm vascular resistance during loading. In contrast, salt loading in salt-sensitive subjects increased this measure (Mark et al., 1975). Vascular compliance of the carotid, femoral, and brachial arteries has also been shown to be reduced in salt-sensitive borderline hypertensives relative to age-matched salt resistance borderline hypertensive subjects and normotensive controls (Draaijer et al., 1993). Importantly, investigations of vascular compliance were undertaken during consistent salt conditions without loading and repletion. There were no differences in cardiac output, blood pressure, and plasma volume between salt resistance and salt-sensitive groups, thus suggesting that hemodynamic differences could not explain differences in arterial compliance but rather this was due to something intrinsic to the vasculature itself. Studies in normotensive individuals have supported a role for the vasculature in SSTHN, wherein salt loading was not shown to lead to differences in external Na^+^ balance, plasma volume, and cardiac output between salt-sensitive and salt-resistant individuals, suggesting other factors outside of renal function lead to blood pressure changes in response to salt loading (Schmidlin et al., 2007). Given the sustained increase in systemic vascular resistance in salt-sensitive individuals that appropriately dissipated in those that were salt-resistant, the authors concluded SSHTN results from systemic vascular dysfunction due to impaired vasodilation in response to increases in dietary NaCl. Importantly, in a follow-up study, salt-resistant subjects demonstrated an efficient response to salt loading with decreased systemic vascular resistance and mean arterial pressure within 24 h. In contrast, systemic vascular resistance did not change throughout the first 3 days of salt loading, whereas mean arterial pressure progressively increased (Schmidlin et al., 2011). Acute studies of salt loading and depletion also suggest a dysregulation of vascular tone in SSHTN that is not attributable to total body autoregulation due to hemodynamic changes that occur during the first 24 h, including impaired vasodilation in response to salt loading and an inability to control total peripheral resistance during depletion in salt sensitive individuals (Laffer et al., 2016).

**FIGURE 1:**
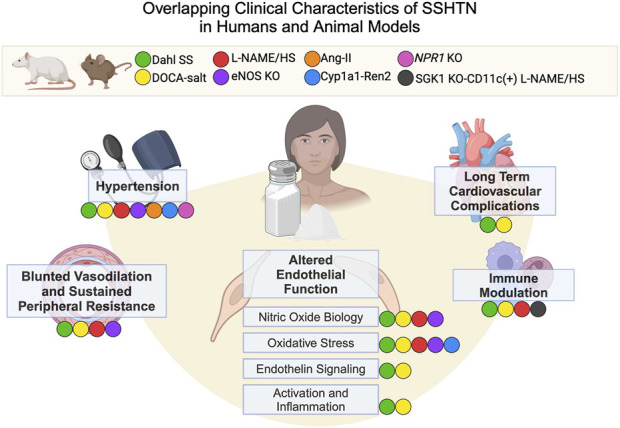
Overlapping Clinical Characteristics of SSHTN in Humans and Animal Models. Animal models and corresponding colors appear in the legend. Clinical characteristics of SSHTN, including hypertension, blunted vasodilation, sustained peripheral resistance, altered endothelial function, immune modulation, and long-term cardiovascular complications, are displayed in bold. Colored labels for animal models appear next to the human clinical characteristics if that model displays the characteristic in question. Dahl SS (Dahl salt-sensitive rats), DOCA-salt (Deoxycorticosterone acetate salt rats and mice), L-NAME/HS (L-NAME administration followed by high salt diet), eNOS KO (eNOS knockout mice), Ang-II (angiotensinogen transgenic mice), Cyp1a1-Ren2 (Cyp1a1-Ren2 transgenic mouse), *Npr1* KO (*Npr1* knockout mouse), SGK1 KO-CD11c (+) L-NAME/HS (mice lacking serum/glucocorticoid kinase 1 in CD11c^+^ cells treated with L-NAME followed by a high salt diet).

These investigations provide a framework for a vaso-dysfunction theory for SSHTN, demonstrating an early failure of vasodilation and increased vascular resistance during salt loading to match normal and salt-resistant controls. Thus, vascular dysfunction is a proposed causative factor in the development of SSHTN (Morris et al., 2016) and has paved the way for mechanistic studies in multiple animal models that mirror the human clinical characteristics (Figure 1).

## Murine models of salt-sensitive hypertension and endothelial dysfunction

Murine models of salt-sensitive hypertension are necessary for understanding this disease’s pathophysiology, underlying mechanisms, and potential therapeutics. The Dahl Salt-Sensitive (SS) rat developed by Dr. Lewis Dahl is one of the most widely used models of salt-sensitive hypertension. This model was developed by selectively breeding Sprague Dawley rats with increased blood pressure responses when challenged with a high-salt diet (Dahl et al., 1962). The Dahl SS rat offers valuable knowledge to study the genetic and physiological components contributing to salt-sensitive hypertension and subsequent endothelial dysfunction and inflammation.

Unlike the Dahl SS rat, no mouse models are currently developed in the same manner of selective breeding for salt sensitivity. However, several transgenic mouse models have been developed to study salt-sensitive hypertension, including the Cyp1a1-Ren2 transgenic mouse, *Npr1* knockout mouse, and angiotensinogen transgenic mouse. Cyp1a1-Ren2 transgenic mice are inserted with the *Ren2* gene under the Cyp1a1 promoter and develop hypertension due to increased salt intake (Howard et al., 2005). *NPR1* knockout mice lack the natriuretic peptide receptor A gene, which affects salt handling in the kidney, resulting in increased blood pressure that is exacerbated by a high-salt diet (Armstrong et al., 2013). Additionally, the angiotensinogen transgenic mice overexpress the angiotensinogen gene, leading to increased levels of angiotensin II (Ang II), a known vasopressor. When fed high-salt diets, these mice exhibit significant blood pressure increases, as observed in SSHTN (Ingelfinger, 2012). While these models are not selectively bred like the Dahl SS rat, they each offer valuable insight into how specific sodium-handling genes play a role in the physiological mechanisms of SSHTN.

Other methods of developing SSHTN in mice and rats involve chemically affecting sodium handling or endothelial nitric oxide (NO) production to induce salt-sensitive hypertension-like phenotype. The deoxycorticosterone acetate (DOCA)-salt model and the Nw-nitro-L-arginine methyl ester (L-NAME) high salt model are commonly used in the field. The DOCA-salt model involves the administration of DOCA and a high-salt diet. Usually, this model is accompanied by a uninephrectomy to elucidate an SSHTN-like phenotype (Dahl et al., 1963; Hua et al., 2024). This model has been instrumental in the pathophysiology mechanisms of hypertension including vascular activity (Lima et al., 2009; Obst et al., 2004), endothelial dysfunction (White et al., 1996), and the role of the sympathetic nervous system (Takeda et al., 1988) in murine SSHTN. Lastly, the L-NAME high salt model of hypertension utilizes the inhibitory effects of L-NAME on nitric oxide synthases, which reduces NO production, resulting in permanent impairment of vascular relaxation and blood pressure regulation. Post administration of L-NAME, usually through drinking water, animals develop hypertension when challenged with a high salt diet (Navaneethabalakrishnan et al., 2024; Majid et al., 2024; Van Beusecum et al., 2019). This model is valuable for studying the interactions between salt intake and NO deficiency (Wang et al., 2020), endothelial dysfunction (Wang et al., 2020), oxidative stress (Zhu et al., 2007), inflammation (Navaneethabalakrishnan et al., 2024; Majid et al., 2024; Van Beusecum et al., 2019; Franco et al., 2013) in the development of hypertension.

## Mechanism of endothelial dysfunction in salt-sensitive hypertension

A key mechanism underlying the development of SSHTN is endothelial dysfunction, which refers to the impaired functioning of the vascular endothelium (Miyoshi et al., 1997; Bragulat et al., 2001). The vascular endothelium is crucial in maintaining vascular tone and health via NO production, promoting vasodilation, and reducing blood pressure. In SSHTN, high salt intake is shown to reduce NO production and increase oxidative stress, resulting in endothelial cell dysfunction and subsequent hypertension (Zhu et al., 2007; Greaney et al., 2012). Understanding the interplay between salt intake, endothelial cell function, and blood pressure regulation is essential for developing effective treatments for salt-sensitive individuals.

Reduced NO production and secretion in Dahl SS rats was first shown by Dr. Matthew Bogehold, who evaluated the influence of endogenous NO on resting microvascular tone (Boegehold, 1992). Dr. Bogehold demonstrated that applying L-NAME induced arteriole constriction only in normotensive Dahl rats and not in Dahl SS rats. Furthermore, he showed that the application of L-arginine, a substrate necessary for activating endothelial nitric oxide synthase (eNOS/NOS3), only induced vasodilation in normotensive Dahl rats. Finally, the application of sodium nitroprusside, an endothelium-independent smooth muscle relaxer, did not induce differential arteriolar vasodilation in Dahl SS rats compared to normotensive. The combination of his findings indicated that endothelial NO mishandling may be responsible for vascular impairment in SSHTN. Utilizing the DOCA-salt model in rats, White et al. (1996) reported similar findings as Dr. Bogehold in the carotid and mesenteric arteries, reporting changes in sensitivity to vasoconstriction via L-NAME administration in DOCA-salt animals compared to controls. Furthermore, they demonstrated that impairment of eNOS activity with L-NAME in control animals increased phenylephrine sensitivity to similar levels as seen in DOCA-salt animals, highlighting the role of endothelial NO in the modulation of vascular smooth muscle contraction in SSHTN. A later study by Hirawa et al. (1996) demonstrated that high-dose administration of cicletanine reduced blood pressure in Dahl SS rats via improved vascular endothelial cell morphology and function measured by histological analysis and increased secretion of endothelial vasodilators such as NO, cyclic GMP, prostacyclin, and PGE_2._

While early studies on the role of endothelial cells in SSHTN explored how the loss of vasodilatory function due to NO impairment in endothelial cells impacted hypertension, d’Uscio et al. explored how endothelin-1 (ET-1), a potent vasoconstricting peptide produced mainly by endothelial cells, regulates vascular tone in Dahl SS rats, finding that the salt sensitive animals had increased plasma ET-1 levels relative to controls (d'Uscio et al., 1997). This was supported by findings from Barton et al. that showed increased aortic tissue levels of ET-1 were inversely correlated with endothelium-dependent relaxation in Dahl SS rats (Barton et al., 1998). *In vitro* experiments in human umbilical vein endothelial cells (HUVECS) have mirrored *in vivo* findings, wherein increased osmolarity due to sodium concentration results in increased ET-1 mRNA levels (Speed et al., 2015).

Antagonism of endothelin receptor A, the vasoconstricting ET-1 receptor, in Dahl SS rats markedly can reduce blood pressure and vascular remodeling of the basilar and mesenteric arteries (d'Uscio et al., 1997). Furthermore, normalized sodium-induced vascular reactivity in the presence of endothelin receptor A antagonism highlights the importance of ET-1/endothelin receptor A mediated endothelial function in salt-sensitive hypertension (Barton et al., 1998). To evaluate whether the vasodilatory endothelin receptor, endothelin receptor B, played a role in endothelial dysfunction in SSHTN, Majane et al. induced SSHTN in rats utilizing the L-NAME high salt model (Majane et al., 2005). Their findings showed that the antagonism of the endothelin receptor B with BQ-788 in combination with L-NAME administration did not intensify blood pressure response compared to L-NAME alone. These results suggest that mishandling of NO was the dominant factor for endothelial dysfunction in SSHTN and most likely occurs in a manner independent of endothelin receptor B (Majane et al., 2005).

Dysregulation of endothelial eNOS activity is known to result in oxidative stress and vascular damage. Kopkan et al. utilized a murine knockout of eNOS to evaluate the effects of a high-salt diet on blood pressure regulation (Kopkan et al., 2010). Results showed that eNOS knockout was sufficient to induce a hypertensive response to a high salt diet, highlighting the importance of NO in SSHTN. Furthermore, reduction of oxidative stress by free radical scavenging with tempol and inhibition of nicotinamide adenine dinucleotide phosphate oxidase resulted in a significant reduction in blood pressure in animals with eNOS knockout, showing the importance of oxidative stress in the development of SSHTN (Kopkan et al., 2010).

## Endothelial cell activation and inflammation in salt-sensitive hypertension

Under physiological conditions, endothelial nitric oxide within the vasculature modulates leukocyte adhesion and migration, this is in response to the tight regulation of NO concentration by eNOS activity (Carreau et al., 2011; Kubes et al., 1991). Under pathological conditions endothelial activation and inflammation are critical processes in perpetuating hypertension (Gallo et al., 2021). Endothelial activation refers to the state in which endothelial cells express adhesion molecules and secrete pro-inflammatory cytokines, which have been well-documented in hypertension (Riou et al., 2007). This leads to immune cells' attraction and subsequent migration to the vascular endothelium. An *in vitro* study by Kumar et al. shows endothelial cells treated with endocan (a secreted peptide during endothelial activation) have reduced eNOS activity, increased inducible NOS (iNOS) expression, and increased nuclear factor kappa B (NFκB) indicating iNOS may be more active than eNOS during endothelial activation (Kumar and Mani, 2021). However, previous studies suggest a more anti-inflammatory role of iNOS in the mediation of endothelial activation reporting effects such as reduced endothelial adhesion molecules, immune cells adherence, and oxidative damage (Hickey et al., 1997; Peng et al., 1998; Hemmrich et al., 2003). The current findings regarding the role of iNOS in the perpetuation of endothelial activation warrant further studies to elucidate its definitive role in endothelial response in salt sensitive hypertension.

Endothelial cell activation has been shown to occur via localized increased levels of interleukin-1-beta (IL-1β) and monocyte chemotactic and activating factor (MCAF) in vascular endothelial in the hearts of Dahl SS rats fed a high salt diet (Shioi et al., 1997). Additionally, Shioi et al. found this pro-inflammatory endothelial state to be associated with increased macrophages in the perivascular area of the vasculature (Shioi et al., 1997). NFκB mediates many of the pro-inflammatory effects, including upregulation of the adhesion molecules intracellular adhesion molecule-1 (ICAM-1) and vascular cell adhesion molecule-1 (VCAM-1) observed during endothelial activation (Kempe et al., 2005; Tabruyn et al., 2009; Fowler et al., 2022). Importantly, the endothelial proteasome has been shown to play a crucial role in activating NFκB (Kempe et al., 2005; Mussbacher et al., 2019). Proteasome inhibition in HUVECs has produced anti-inflammatory effects, as documented in Ludwig et al., including reducing adhesion molecule expression and resistance to tumor necrosis factor-alpha (TNF-α)-induced endothelial activation (Ludwig et al., 2009). The role for TNF-α-induced endothelial activation was further divulged by Cai et al., wherein the authors demonstrated that knockout of TNF-α in DOCA-salt mice resulted in decreased blood pressure and less vascular remodeling (Cai et al., 2020). Furthermore, they showed that TNF-α knockout increased eNOS expression in the aortic tissue and reduced monocyte chemoattractant protein-1 (MCP-1) expression and F4/80 positive macrophages (Cai et al., 2020). These findings from Ludwig and Cai indicate that TNF-α signaling mediates oxidative stress and endothelial activation in SSHTN. Additionally, adaptive immune cells such as CD4^+^ T cells have been shown by Linghui et al. to be regulated by activated endothelial cells in response to the catecholamine norepinephrine (Xu et al., 2018), a catecholamine known to be significantly elevated in humans with salt-sensitive hypertension (Fujita et al., 1980; Campese et al., 1982; Gill et al., 1988). More specifically, Lingui demonstrated that pretreatment of endothelial cells with norepinephrine resulted in increased secretion of cytokines IL-17A and IL-6. Additionally, when cocultured with CD4^+^ T cells, norepinephrine treated endothelial cells promoted CD4^+^ T cell class switching to pro-inflammatory Th17 phenotype (Xu et al., 2018), indicating the catecholamines may play a role in endothelial activation and subsequent immune regulation in salt-sensitive hypertension.

The potent vasoconstrictor 20-hydroxyeicosatetraenoic acid (20-HETE), a metabolite of cytochrome P4504A (CYP450), has been implicated in the development of hypertension and renal injury in Dahl SS Rats (Levere et al., 1990). *In vitro* studies by Imig et al. utilized Sprague Dawley rat kidneys demonstrated that inhibition of CYP450 activity resulted in increased vasodilation of the proximal and distal afferent arterioles and loss of glomerular capillary pressure autoregulation indicating a role of CYP450 metabolites in vasoconstriction responses of the afferent arterioles (Imig et al., 1994). Studies utilizing human umbilical vein endothelial cells (HUVECs) observed increases in the adhesion molecule ICAM-1 and pro-inflammatory cytokine secretion of IL-4, IL-6, IL-8, and IL-13 in response to 20-HETE treatment indicating a role for 20-HETE in the propagation of endothelial activation/inflammation (Ishizuka et al., 2008). *In vivo* studies by Cheng et al. demonstrated that endothelial specific genetic knock-in of human CYP4F2 resulted in increased 20-HETE protein levels in the renal interlobar arteries and aortas and increased secretion of 20-HETE from renal endothelial cells. Additionally, increased 20-HETE expression from CYP4F transgenic renal endothelial cells coincided with increased oxidative stress markers and superoxide levels compared to wild-type cells. Furthermore, they found increased vasoconstriction responses in aortas isolated from CYP4f2 transgenic animals compared to wild-type controls when treated with phenylephrine (Cheng et al., 2014). The combination of these results indicates that 20-HETE and CYP450 family enzymes play a role in the development of endothelial activation and dysfunction via pro-inflammatory cytokine regulation and NO bioavailability.

**FIGURE 2:**
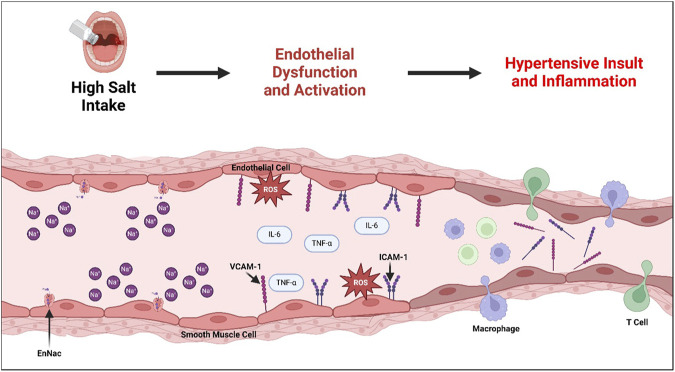
Development of Endothelial Dysfunction and Subsequent Hypertension and Associated Inflammation in Salt-Sensitive Hypertension. Consumption of a high-salt diet has been shown to upregulate the activity of endothelial sodium transporters (ENaC), which plays a role in developing subsequent endothelial dysfunction. Endothelial dysfunction is marked by increased reactive oxygen species (ROS), often due to impaired eNOS activity. Furthermore, endothelial dysfunction leads to the expression of cytokines, including TNF-α and IL-6, and adhesion molecules ICAM-1 and VCAM-1, resulting in a pro-inflammatory endothelial activation state. Prolonged endothelial dysfunction and activation narrows the arteries due to impaired endothelium-dependent relaxation, creating a hypertensive insult and associated vascular inflammation and immune cell recruitment.

While we have highlighted the complexity of endothelial responses to SSHTN as experimentally shown in both *in vivo* and *in vitro* models (Figure 2), further studies are needed to understand the interactions and contributions of vascular dysfunction, NO mishandling, iNOS activity, norepinephrine, and endothelial activation on subsequent hypertension and inflammation in response to increased concentrations of dietary salt.

## Sex differences in salt-sensitive hypertension

The epidemiology of SSHTN largely suggests an association with sex (Chen, 2010; Schmidlin et al., 2011; He et al., 2009; Vollmer et al., 2001; Bray et al., 2004; Parksook et al., 2022; Shukri et al., 2018; Elliott et al., 1989), with few exceptions (Weinberger et al., 1986; Ishibashi et al., 1994). Gender differences in blood pressure responses to dietary salt reveal females have increased salt sensitivity, with some estimates at 30% higher salt sensitivity in females than males (Shukri et al., 2018). A dietary intervention study, Dietary Approaches to Stop Hypertension (DASH)-Sodium Trial, demonstrated that lowering salt intake unvaryingly resulted in the overall lowering of blood pressure, especially in hypertensives and older individuals (Vollmer et al., 2001). Interestingly, this dietary intervention revealed that relative to men, women had a greater decrease in systolic blood pressure with a reduction in salt intake. However, this decrease was not reflected in diastolic BP between the sexes (Vollmer et al., 2001; Bray et al., 2004).

While it is largely known that the prevalence and severity of hypertension increases in women after menopause (Trémollières et al., 1999), data further supports an association between salt sensitivity and changes to the hormonal profile (Parksook et al., 2022; Tominaga et al., 1991; Schulman et al., 2006; Manosroi et al., 2017). This link between hormones and salt sensitivity appears to be independent of the effect of aging, as surgical menopause in healthy premenopausal women has been shown to double the prevalence of SSHTN (Schulman et al., 2006). Further, genetic variations in the estrogen receptor-2 gene (*Esr2*) highlight the risk of altered hormonal profiles to SSHTN prior to menopause, with women carrying the risk allele having a greater risk for SSHTN (Manosroi et al., 2017).

Changing hormonal profiles has been linked to an increased risk of SSHTN through endothelial dysfunction through aldosterone mediation (Shukri et al., 2018; Manosroi et al., 2017; Faulkner et al., 2018; Faulkner et al., 2020; Faulkner et al., 2021), decreased NO bioavailability (Schmidlin et al., 2011; Faulkner et al., 2020; Faulkner et al., 2021; Virdis et al., 2000), Ang II modulation (Harrison-Bernard et al., 2003). Murine studies with high salt diets demonstrate that females develop higher blood pressure, paired with elevated aldosterone/renin ratios and impaired endothelial relaxation relative to males (Faulkner et al., 2018). This endothelial dysfunction was linked to mineralocorticoid receptors, which restored female blood pressure and endothelial function when inhibited. The same group further linked increased aldosterone to endothelial dysfunction through reduced NO availability through mineralocorticoid mechanisms (Faulkner et al., 2020; Faulkner et al., 2021). Surgical deficiency of estrogens has been shown to increase salt sensitivity in Dahl SS and spontaneously hypertensive rats through activation of Ang II, thus lowering the availability of endothelial NO (Harrison-Bernard et al., 2003). Importantly, comparative studies in Dahl SS rats between males, intact females, and ovariectomized females show that sodium retention does not contribute to sex differences within these models, supporting the possible pressor effect of the endothelium on SSHTN development (Hinojosa-Laborde et al., 2000).

Therapeutically, hormone replacement therapy improves endothelial function in the forearm resistance arteries (Sanada et al., 2002), with greater benefit to hypertensive women relative to their normotensive counterparts (Higashi et al., 2001). The protective effect of hormone replacement has also been demonstrated with reduced plasma levels of the vasoconstrictive ET-1 (Ylikorkala et al., 1995). However, the dichotomy in immune activation and pro- and anti-inflammatory cytokine signaling in SSHTN between sexes must be further understood to improve therapeutics for both men and women.

Mechanistic *in vivo* studies of immune differences between the sexes reveal the magnitude of such dichotomy. Germline deletion of a cell surface protein involved in the innate immune system, CD14, worsened hypertension and renal injury in Dahl salt-sensitive females but not males, which was reversed with ovariectomy and the removal of female sex hormones (Fehrenbach et al., 2021). Activation of the adaptive immune response is also linked to hormones, wherein the activation of T-cells in hypertension involves, like in the endothelium, the aldosterone-mineralocorticoid axis (Sun et al., 2017). Differences in T-cell infiltration further highlight the importance of sex in SSHTN. Dahl SS male and female rats challenged to a high salt diet had a reduction in blood pressure and proteinuria when genetically deleted of T cells (Abais-Battad et al., 2024). However, the adoptive transfer of splenocytes into these rats showed that hypertension was augmented if the recipient or T-cells transferred were male (Abais-Battad et al., 2024). Similar adoptive transfer studies of male T cells into Rag-1 (^−/−^) male and female mice demonstrated that females were protected from Ang II-mediated increases in hypertension seen in their male counterparts (Pollow et al., 2014). Though this study lacked the appropriate female-to-female transfer to understand the attribution of donor sex on hypertension development fully, it does suggest a role for sex-specific immune differences in models beyond salt loading. Importantly, protection against T cell-mediated Ang II hypertension is abrogated in menopause, suggesting a hormonal influence on proinflammatory responses (Pollow et al., 2019).

## Conclusion and perspectives

Numerous clinical studies and murine models have contributed to the critical understanding of the mechanisms of developing endothelial and vascular dysfunction in SSHTN. We have discussed the key clinical studies, murine models, and mechanisms contributing to endothelial dysfunction, activation, and pathophysiological signaling that promote deleterious elevations in blood pressure in response to a high salt intake. Vascular and endothelial dysfunction has always been a key mechanism in elevated blood pressure in salt sensitivity. However, recent studies have demonstrated the key role of endothelial cell activation and dysfunction in orchestrating inflammation and elucidating key sex-dependent mechanisms. Ideally, novel cell-specific therapies would prevent endothelial cell dysfunction and activation, thus preventing the generation of reactive oxygen species, increasing NO bioavailability, and preventing the transmigration of immune cells to end-organs. The design of these novel therapies could target key proteins and signaling mechanisms that contribute to endothelial cell dysfunction to modulate the key mechanisms discussed in the above review. Targeted delivery of these therapies to the endothelial cells could alter vascular tone and physiology, endothelial activation status, and prevent subsequent immune cell infiltration to target organs. Further studies, specifically elucidating the sex-dependent endothelial and vascular mechanisms in SSHTN and translational studies targeting endothelial cells, are needed to delineate critical mechanisms in patients with SSHTN.
